# Effects of Hyaluronic Acid in the Surgical Treatment of Periodontitis: A Systematic Review

**DOI:** 10.3290/j.ohpd.c_2760

**Published:** 2026-08-26

**Authors:** Zocko Ange Désiré Pockpa, Camille Bechina, Marie Boutet, Zahi Badran, Assem Soueidan, Xavier Struillou

**Affiliations:** a Zocko Ange Désiré Pockpa Assistant Professor of Periodontology, Félix Houphouët-Boigny University, Department of Periodontology, Dental College, Abidjan, Côte d’Ivoire. Concept and study design, data collection, performed research, manuscript drafting.; b Camille Bechina Assistant Professor of Periodontology, Nantes University, CHU Nantes, Department of Periodontology, Nantes Dental College, F-44000 Nantes, France. Performed research, reviewed and approved the final manuscript.; c Marie Boutet Dentist, Nantes University, CHU Nantes, Department of Periodontology, Nantes Dental College, F-44000 Nantes, France. Concept and study design, performed research.; d Zahi Badran Professor of Periodontology, College of Dental Medicine, University of Sharjah, Sharjah, United Arab Emirates. Critical proofreading, discussion, reviewed and approved the final manuscript.; e Assem Soueidan Professor of Periodontology, Nantes University, CHU Nantes, Department of Periodontology, Nantes Dental College, F-44000 Nantes, France. Project supervision, study design, synthesis of results, reviewed and approved the final manuscript.; f Xavier Struillou Professor of Periodontology, Félix Houphouët-Boigny University, Department of Periodontology, Dental College, Abidjan, Côte d’Ivoire. Project supervision, study design, synthesis of results, reviewed and approved the final manuscript.

**Keywords:** hyaluronic acid, inflammatory diseases, periodontitis, surgical periodontal therapy, wound healing

## Abstract

**Purpose:**

Hyaluronic acid (HA) is a naturally occurring polymer synthesised endogenously in the human body, with distinctive physicochemical and biological properties such as excellent biocompatibility and biodegradability. These characteristics have led to extensive research into its therapeutic potential, particularly in the management of inflammatory diseases. This systematic review aimed to evaluate the effects of HA on clinical outcomes in the surgical treatment of periodontitis.

**Methods and Materials:**

This review was registered with PROSPERO (CRD42024493296) and conducted according to Preferred Reporting Items for Systematic Reviews and Meta-Analyses (PRISMA) 2020 guidelines. A systematic search was performed in PubMed, Cochrane, and Scopus up to September 2025. Randomised controlled trials assessing HA clinical outcomes in the surgical treatment of periodontitis were included.

**Results:**

Nine RCTs including a total of 343 patients were analysed. Sample sizes ranged from 14 to 60 participants, with follow-up periods between 3 and 24 months. The adjunctive use of HA in the surgical treatment of periodontitis led to consistent improvements, with probing depth (PD) reductions of 2.0–3.5 mm and CAL gains of 2.0–3.8 mm after 6–12 months. Radiographic evaluations also showed greater bone fill in intrabony defects.

**Conclusion:**

Within the limits of available randomised controlled trials, HA shows potential as an adjunct in the surgical management of periodontitis. Although reported improvements in clinical parameters are encouraging, evidence remains heterogeneous and requires confirmation through standardised, long-term studies.

Hyaluronic acid (HA) is a naturally occurring polymer synthesised endogenously in the human body, with distinctive physicochemical and biological properties such as excellent biocompatibility and biodegradability.^10^ These characteristics have led to extensive research into its therapeutic potential, particularly in the management of inflammatory diseases.^16,24^

In recent years, HA has gained increasing attention in the treatment of periodontitis and peri-implantitis^14,23^ Periodontitis is a chronic multifactorial inflammatory disease initiated by dysbiotic biofilms, resulting in progressive destruction of tooth-supporting structures.^13,18^ Clinically, it is characterised by clinical attachment loss (CAL), alveolar bone resorption, periodontal pocket formation, and bleeding on probing (BOP).^13,18^ When the indication is well established, the use of biological products in treatment would provide additional clinical benefits.^5,26^ However, the effects of these regenerative approaches on long-term bone reconstruction remain controversial.^32^ Within periodontal tissues, HA is abundantly distributed in the gingiva and periodontal ligament, where it contributes to tissue integrity, wound healing, and repair.^7^ During inflammation, however, the production of hyaluronidase leads to degradation of endogenous HA, which provides the rationale for its therapeutic supplementation.^10^

At the molecular level, HA interacts with cell surface receptors such as CD44 and RHAMM, influencing fibroblast migration, angiogenesis, and cytokine modulation. It contributes to the regulation of extracellular matrix (ECM) turnover, reduces oxidative stress, and supports macrophage polarisation toward a regenerative phenotype. These biological mechanisms form the scientific basis for using HA as an adjunctive biomaterial in the treatment of periodontitis.

Owing to its anti-inflammatory, antimicrobial, and wound-healing properties, exogenous HA has emerged as a promising adjunctive agent in periodontal treatment. Previous studies suggest that HA may enhance soft tissue regeneration, stimulate angiogenesis, modulate inflammatory responses,^2,14^ and improve patient-reported outcomes such as pain relief and comfort. However, despite these encouraging findings, the extent and consistency of HA’s effects remain variable across studies. Further clarification is required to determine whether its clinical improvements are statistically and biologically significant.

This systematic review evaluates the adjunctive effects of HA in the surgical treatment of periodontitis, focusing on clinical outcomes (probing depth [PD] reduction and CAL gain) and biological effects to clarify its therapeutic efficacy and identify evidence gaps.

## METHODS AND MATERIALS

### Protocol and Registration

The protocol for this review was registered in the Prospective International Registry of Systematic Reviews (PROSPERO)^8^ under ID CRD42024493296 and followed the 2020 Preferred Reporting Items for Systematic Reviews and Meta-Analyses (PRISMA) guidelines.^17^

### Eligibility Criteria

The PICOS structure was used to define the inclusion criteria and formulate the research question. Studies published in English that met the following criteria were included:

1. Participants (P): Patients with periodontitis stage III/IV, grade B/C.
2. Intervention (I): Surgical periodontal therapy with HA.
3. Comparison (C): Comparisons of the effects of HA on periodontal parameters between the test group (which received HA) and the control group (which did not receive HA).
4. Outcomes (O): The primary outcome was PD reduction; The secondary outcomes included clinical attachment level (CAL), bleeding on probing (BOP), bone gain, and microbiological or inflammatory markers.
5. Study design (S): Only randomised controlled trials (RCTs) were selected.

### Search Strategy

**Fig 1:**
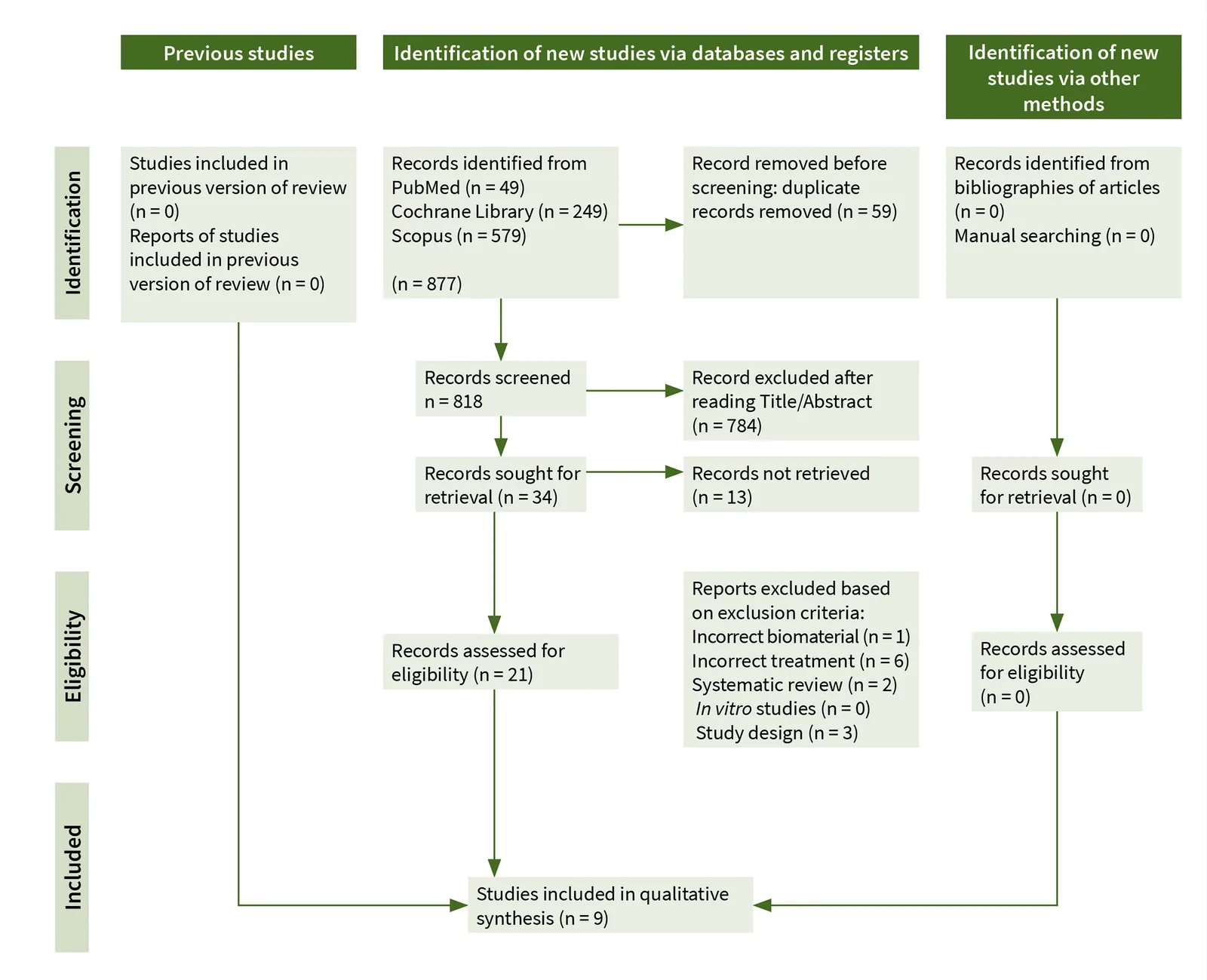
PRISMA flow diagram.

A comprehensive literature search was performed in PubMed, Cochrane, and Scopus up to September 2025, without year restriction, in order to capture all available studies evaluating HA in periodontology. The search combined keywords and Medical Subject Headings (MeSH) using various equations as summarised in Table 1. In addition, manual searches were performed in reference lists of relevant articles and in specialised journals to identify potential studies not captured by electronic search. All references were managed with Zotero, and duplicate records were removed before screening (Fig 1).

**Table 1:** Databases and search terms

Databases (filters applied)	Search terms
PubMed (randomised controlled trial)	((Periodontitis) OR (open flap debridement) OR (periodontal surgery) OR (intrabony defect) OR (intrabony lesion) OR (intraosseous defect) OR (periodontal defect) OR (periodontal lesion)) AND ((hyaluronic) OR (hyaluronic acid) OR (hyaluronan))
Cochrane library (all text)	((Periodontitis) OR (open flap debridement) OR (periodontal surgery) OR (intrabony defect) OR (intrabony lesion) OR (intraosseous defect) OR (periodontal defect) OR (periodontal lesion)) AND ((hyaluronic) OR (hyaluronic acid) OR (hyaluronan))
Scopus (dentistry, article, english, humans)	((Periodontitis) OR (open flap debridement) OR (periodontal surgery) OR (intrabony defect) OR (intrabony lesion) OR (intraosseous defect) OR(periodontal defect) OR (periodontal lesion)) AND ((hyaluronic) OR (hyaluronic acid) OR (hyaluronan))


### Selection Process

The research and article selection processes were carried out independently by two authors (BM and PZ). The two reviewers manually searched for additional published papers that could meet the eligibility criteria of this study. After removing non-relevant articles by screening titles and abstracts, the full-text articles were obtained and evaluated separately by independent reviewers (PZ, BM). In case of disagreement between reviewers, inclusion or exclusion of the articles was determined by a third reviewer (XS) (Fig 1).

### Data Collection Process

The investigators extracted data from the included articles and organised them according to the following criteria: author, year, country, study design, participant characteristics, intervention details, follow-up duration, clinical parameters, outcomes, and conclusions.

### Study Risk of Bias Assessment

**Fig 2:**
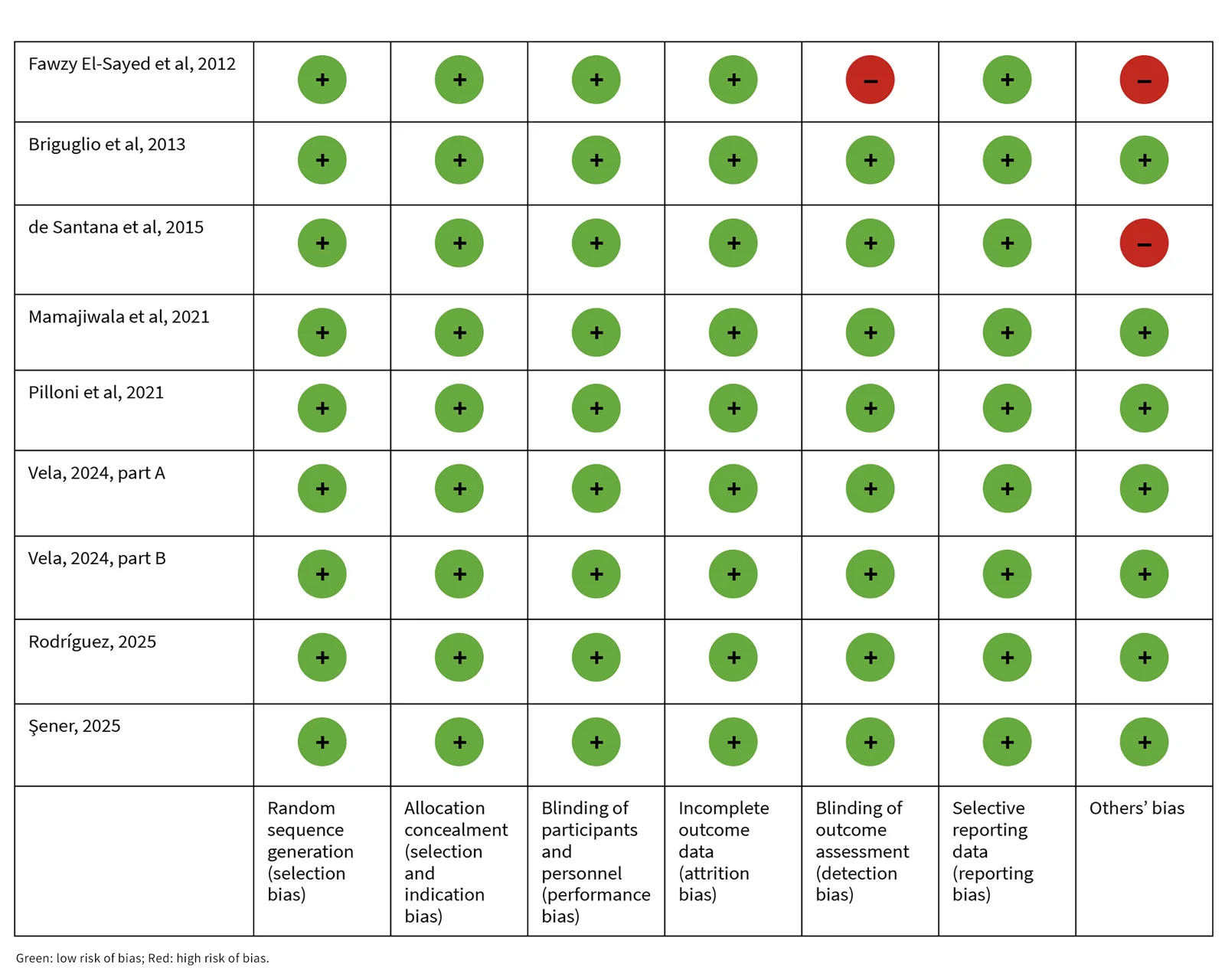
Risk of bias of included studies.

The risk of bias assessment was performed with Cochrane’s Collaboration tool for assessing the risk of bias in RCTs. Two authors (BM and PZ) assessed and discussed the risk of bias in the included studies. The risk of bias level was determined to be low, unclear, or high according to the following criteria: (1) generation of the randomisation sequence (selection bias); (2) concealment of the allocation (reporting bias); (3) blinding of the investigators and the participants (confusion bias); (4) blinding of the evaluation of the results (performance bias); (5) management of missing data (attrition bias); (6) selection of the reporter; and (7) other types of bias (Fig 2).

## RESULTS

### Study Selection

The bibliographic search identified a total of 877 articles from the databases. After removing 59 duplicates, 818 records remained for screening. Following title and/or abstract screening, 784 articles were excluded, and 13 additional records were excluded because the full texts could not be retrieved. Ultimately, 21 full-text articles were assessed for eligibility, and 9 studies met the inclusion criteria and were included in the systematic review (Fig 1).

### Description of the Included Studies

Table 2 summarises the characteristics of the included RCTs.

**Table 2:** Characteristics of the studies

Author (Year)	Country	Participants (n)	Inclusion criteria	Mean age (years)	Study design	Test intervention	Control intervention
Fawzy El-Sayed et al. (2011)	Egypt	14	CP cases with ≥ 4 interproximal sites with moderate to deep IBD (≥ 3 mm) and PD > 5 mm on premolars or molars	NR	SmRCT	MWF + 0.8% HA	MWF + placebo
Briguglio et al. (2013)	Italy	40	Subjects with advanced generalised CP, angular two-wall infrabony defect in the interproximal area (PD ≥ 7 mm; CAL > 7 mm)	45 ± 8.2	RCT	OFD + HA	OFD alone
De Santana et al. (2015)	Brazil	30	Adult with CP and IBD > 4 mm, PD > 6 mm	49.2 ± 3.16	RCT	MPP + rhFGF-2/HA	MPP alone
Mamajiwala et al. (2021)	India	20	Subjects with PC having ≥ 2 contralateral IBD ≥ 3 mm and PD ≥ 5 mm.	39.9 ± 4.18	SmRCT	OFD + 0.8% HA	OFD + placebo
Pilloni et al. (2021)	Italy	32	Subjects with stage 3 generalised periodontitis having ≥ 2 nonadjacent teeth showing one residual pocket with PD ≥ 5, without mobility and furcation involvement.	41.47 ± 9.25	RCT	HA (mixture of cross-linked 1.6% + natural 0.2%)	EMD
Vela et al. Part A (2024)	Romania	54	Patients with periodontitis stages II/III, grades A/B, and ≥ 2 suprabony defects	NR	RCT	xHyA	EMD
Vela et al, Part B (2024)	Romania /Italia	60	presence of 2–3 walls IBD ≥ 3 mm and interproximal PPD ≥ 6 mm;	NR	multicentric RCT	OFD + HA	OFD alone
Rodríguez-A et al (2025)	Spain	53	Presence of a single IBD with PD ≥ 6 mm and a defect component ≥ 3 mm.	NR	RCT	xHyA	EMD
Şener et al (2025)	Turkey	18	Patients diagnosed with Stage III Grade B or C periodontitis.	NR	SmRCT	OFD + 0.8% HA gel	OFD alone
NR: not reported; MWF = modified Widman flap; OFD = open flap debridement; HA = hyaluronic acid; xHyA = cross-linked hyaluronic acid; EMD = enamel matrix derivatives; MPP = modified papilla preservation; PD = probing depth; CAL = clinical attachment level; GR/REC = gingival recession; PI = Plaque Index; GI = Gingival Index; BOP = bleeding on probing; CEJ = cemento-enamel junction; PAL = probing attachment level; GCF = gingival crevicular fluid; ABL: alveolar bone loss; IBD: intrabony defect; TOS/TAS = total oxidant/antioxidant status; RCT: randomised controlled trial; SmRCT: split-mouth randomised clinical trial; CP: chronic periodontitis.							

A total of nine RCTs involving 343 patients were analysed (Fig 2). These studies evaluated the adjunctive use of HA in surgical periodontal procedures such as open flap debridement (OFD) and modified Widman flap (MWF), with follow-up periods ranging from 3 to 24 months. The HA formulations used included 0.8% HA gel^9,15,30^ and cross-linked HA,^3,20,25,28,32^ applied locally or in combination with enamel matrix derivatives (EMD).^33^

### Study Outcomes

Across studies, the adjunctive application of HA consistently improved key clinical outcomes compared to surgery alone. Mean probing depth (PD) reductions ranged from 2.0 to 6.0 mm, while CAL gains ranged from 2.5 to 5.0 mm. These improvements appeared as early as 3 months and remained stable up to 24 months. Several studies also reported reductions in gingival recession (GR) and BOP, along with decreased levels of local inflammatory biomarkers (IL-6, IL-8, IL-10, IL-17).^30^ Radiographic analyses revealed significant bone regeneration, with CAL gains ≥ 4 mm in up to 48% of treated sites.^25^ These findings align with those of Vela et al,^31,32^ who demonstrated superior clinical and radiographic outcomes with cross-linked HA compared to EMD in intrabony defects after 6 months.

### Analysis of the Risk of Bias

Most studies demonstrated an overall low-to-moderate risk of bias. A few showed higher attrition rates, mainly due to loss to follow-up or antibiotic use. Blinding procedures were inconsistently applied, although randomisation methods and allocation concealment were generally adequate (Fig 1).

## DISCUSSION

Multiple types of adjunctive therapeutic strategies have been established during the last decades in periodontology for better clinical outcomes, mostly related to usage of biomaterials such as bone substitutes, EMD, barrier membranes and growth factors.^6,11,29^ HA, due to its hygroscopic properties, anti-inflammatory and antimicrobial effects, also demonstrates a capacity to absorb growth factors and promote tissue regeneration.^1,4^

Several recent studies have confirmed these properties, demonstrating improved soft tissue healing, reduction of inflammatory mediators, and favourable clinical attachment gains when HA is used as an adjunct.^12,16^ In this systematic review, we analysed RCTs that assessed the effects of HA on clinical outcomes in the surgical treatment of periodontitis.

Across the included studies, adjunctive use of HA was associated with reductions in PD and improvements in CAL. These results suggest a potential clinical effect of HA; however, variability in formulations, protocols, and study designs limits the generalisation of these findings.

The use of HA as an adjunct to surgical periodontal therapy has been associated with consistent improvements in key clinical parameters, particularly PD and CAL. These improvements are likely related to HA’s biochemical and biophysical properties.^4^ Its hygroscopic nature promotes tissue hydration and viscoelasticity, providing a favourable environment for fibroblast migration, proliferation, and differentiation. In addition, HA exhibits anti-inflammatory effects by modulating cytokines such as IL-1β and TNF-α, which helps reduce connective tissue breakdown and supports periodontal regeneration.^4,31^ Its antimicrobial properties may also contribute to reducing the pathogenic bacterial load, further enhancing healing.

The recent RCTs (Table 1) reinforced these observations, showing that adjunctive HA therapy was associated with greater PD reduction, improved CAL gains, and enhanced early wound healing compared with surgery alone.^9,3,28,15,20,31,32,25,30^ Mechanistic studies have also demonstrated that HA promotes extracellular matrix remodelling, collagen maturation, and angiogenesis, which may contribute to accelerated wound healing and improved tissue quality.^20,22^ Some studies combined HA with growth factors, suggesting potential synergistic interactions that could enhance regenerative outcomes beyond those achieved with surgery alone.^9,3,15,20,25,28,30–33^

Clinically, these biological mechanisms correspond to measurable PD reductions and CAL gains, and in some cases, decreased BOP, although BOP data were inconsistently reported. Recently, Pilloni et al^22^ provided a comprehensive overview of the clinical applications of HA in periodontal therapy. Their review encompassed the full spectrum of HA use, including early wound healing, adjunctive applications in non-surgical therapy, regenerative surgical management of intrabony defects, and mucogingival procedures for root coverage. The clinical effects of HA in periodontal therapy likely result from a combination of biological and physicochemical mechanisms acting throughout the wound-healing cascade. Due to its high molecular weight and viscoelastic properties, HA maintains tissue hydration, lubrication, and structural integrity, thereby creating a favourable microenvironment for tissue repair and regeneration.^4,31^

At the cellular level, HA facilitates fibroblast and keratinocyte migration, promotes proliferation of periodontal ligament and gingival cells, and serves as a biocompatible scaffold for the attachment and controlled release of growth factors.^4,31^ This scaffolding function contributes to the regulation of signalling pathways involved in extracellular matrix (ECM) remodelling and supports wound stability.^4,31^

Its anti-inflammatory activity is mainly mediated through modulation of cytokine networks such as IL-1β and TNF-α and inhibition of prostaglandin and matrix metalloproteinase production, leading to a reduction in connective tissue degradation. In addition, HA displays antimicrobial activity by interfering with bacterial adhesion, biofilm maturation, and colonisation by key periodontopathogens such as *Porphyromonas gingivalis*.^4,31^ HA can enhance collagen fibrillogenesis, angiogenesis, and ECM remodelling, thereby accelerating soft tissue healing, promoting neovascularisation, and improving vascular supply to periodontal tissues.^27^ These biological processes contribute to improved oxygenation, nutrient transport, and stabilisation of newly formed tissues.^19^ When used in combination with growth factors, EMD, or bone substitutes, HA may also exert synergistic regenerative effects by potentiating osteogenic differentiation, bone matrix deposition, and long-term tissue stability.^24^ Such multimodal interactions suggest that HA could act as a supportive biomaterial within complex regenerative protocols, rather than as a stand-alone therapeutic solution for periodontitis.

### Limitations and Recommendations

This systematic review aimed to evaluate the effects of HA on clinical outcomes in the surgical treatment of periodontitis. HA can be recommended as an adjunct in the surgical treatment of sites with moderate to deep infraosseous defects (≥ 3 mm). The mean attachment gain obtained in studies ranged from 0.5 to 5.1 mm, while the PD reduction ranged from 1.6 to 5.3 mm. Data remain limited and inconclusive for supraosseous defects and Class II furcations.

Several limitations should be considered when interpreting the findings. First, most included RCTs were conducted on small samples, which limits statistical power and restricts the generalisability of the results. Second, there was substantial heterogeneity among studies regarding surgical protocols, HA formulations and concentrations, and follow-up durations. Such variability reduces the comparability of studies and weakens the overall strength of the conclusions. Third, reporting of BOP was inconsistent or incomplete in several trials, preventing a thorough evaluation of this important clinical parameter. Moreover, most studies provided only short- to medium-term data, leaving uncertainty about the long-term stability of the improvements associated with adjunctive HA. The four most recent RCTs offer additional information and broader clinical contexts, but heterogeneity remains an important limitation (Table 3).

**Table 3:** Summary of key findings and follow-up duration in periodontitis studies

Author (year)	Follow-up (months)	Primary outcome	Secondary outcomes	Main results
Fawzy El-Sayed et al (2011)	3–6	Change in CAL	GR, PD, BOP, and PI	Statistically significant differences (*P* < 0.05) were noted between the test and the control sites at 3 and 6 months, where the test sites showed greater gain in CAL and reduction in GR values. No statistically significant differences regarding PD, BOP, and PI values (*P* > 0.05).
Briguglio et al (2013)	12–24	Change in CAL	PD, BOP, PI	Test defects shows a mean CAL gain of 1.9 ± 1.8 mm, while the control defects yielded a significantly lower gain of 1.1 ± 0.7 mm (*P* < 0.05) ; PD reduction was also significantly higher in the test group (1.6 ± 1.2 mm) than in the control group (0.8 ± 0.5 mm) (*P* > 0.05).
De Santana et al (2015)	12	Change in CAL	Bone gain	Combination of bioactive substances composed of rhFGF-2/HA applied in periodontal intrabony defects results in significantly more PD reduction, CAL and bone gains (*P* < 0.01).
Mamajiwala et al (2021)	6–12	Change in CAL	PD, bone defect fill, GR	After 12 months, the test group showed significantly greater CAL gain (5.1 ± 1.2 vs 4.05 ± 1.19 mm) and bone defect fill (DF) (5.67 ± 2.01 vs 4.49 ± 1.78 mm) compared to the control group (*P* < 0.01). Mean PD reduction in the test group (5.3 ± 1.2 vs 4.35 ± 0.81mm) was statistically significant compared to the control group at 12-month period (*P* < 0.01).
Pilloni et al (2021)	12–18–24	Changes in PD	REC, CAL, BOP, mSBI, PI	The test group showed better results in terms of PD reduction (2.08 ± 1.24 vs. 1.94 ± 1.19, *P* = 0.533) and sites with PD ≤ 4 mm (38/50 vs 35/50, *P* = 0.499), although not statistically significant. Similarly, CAL gain was comparable between groups (test : 0.50 ± 1.85 vs. control : 0.36 ± 1.80, *P* = 0.700). Significantly higher reduction in mSBI was recorded in the test group only in sites with baseline PD ≥ 6 mm (*P* = 0.004).
Vela et al, Part A (2024)	6	Change in CAL	PD, GR, BOP, FMPS, FMBS	In the control and test groups, the mean CAL gain was statistically significant in the intragroup comparison (*P* < 0.001). 48.1% of test sites showed a CAL gain ≤ 2 mm compared with 33.3% of control sites. The mean PPD reduction was statistically significant in the intragroup comparison in both groups (*P* < 0.001). The mean REC increase was similar in the two groups: 1.04 ± 1.29 mm vs 1.11 ± 1.22 mm (test vs control).
Vela et al. Part B (2024)	12	Change in CAL	PD, GR, FMPS, FMBS, REC, BOP	The mean CAL gain was statistically significantly different (*P* < 0.001) in the test group compared with the control group (3.06 ± 1.13 mm vs 1.44 ± 1.07 mm). PD reduction of test group measurements (3.28 ± 1.14 mm) versus the control group measurements (2.61 ± 1.22 mm) were statistically significant (*P* = 0.032).
Rodríguez-A et al. (2025)	6-12-18	Change in CAL	PD, BOP, GR, FMBS FMPS, tooth mobility	At 6, 12, and 18 months after surgery demonstrated significant improvements in CAL, PD, REC,BOP for both groups compared with baseline (*P* < 0.001). HA group demonstrated a CAL gain ≥ 4 mm at 18 months, observed in 13 of 27 (48.1%) defect sites.
Şener et al. (2025)	6	Change in CAL	PD, BOP, GCF biomarkers	The application of 0.8% HA gel addictive with OFD provides significant improvements in CAL, PD, positive results in GCF IL-10 and IL-17 A levels in patients with Stage III periodontitis compared to OFD alone (*P* < 0.001).
NR: not reported; MWF = modified Widman flap; OFD = open flap debridement; HA = hyaluronic acid; xHyA = cross-linked hyaluronic acid; EMD = enamel matrix derivatives; MPP = modified papilla preservation; PD = probing depth; CAL = clinical attachment level; GR/REC = gingival recession; PI = Plaque Index; GI = Gingival Index; BOP = bleeding on probing; CEJ = cemento-enamel junction; PAL = probing attachment level; mSBI = modified sulcus bleeding index; FMPS = full-mouth plaque score; FMBS: full-mouth bleeding score; GCF = gingival crevicular fluid; RCT: randomised controlled trial. SmRCT: split-mouth randomised clinical trial.				

Given these limitations, caution is needed when interpreting the available evidence. More standardised and methodologically rigorous research is required to strengthen conclusions on the clinical benefits of HA in periodontal surgery. To address these gaps, future studies should:

Conduct larger, multicenter RCTs with sufficient statistical power and longer follow-up to confirm the persistence of clinical benefits.
Standardise HA formulations, concentrations, and application protocols to improve reproducibility and facilitate comparisons across studies.
Examine dose–response effects and evaluate long-term clinical, microbiological, and radiographic outcomes.
Assess potential synergistic combinations of HA with growth factors, biomaterials, or other bioactive agents to enhance regenerative and anti-inflammatory effects.
Include patient-centred outcomes such as postoperative pain, healing comfort, and quality of life to complement clinical measures and reinforce the clinical relevance of future research.

## CONCLUSION

This systematic review suggests that HA may represent a promising adjunct in the surgical treatment of periodontitis. Across RCTs, HA was associated with reductions in PD and gains in CAL, outcomes that could be linked to its anti-inflammatory, antimicrobial, and wound-healing properties. However, evidence regarding BOP, microbiological outcomes, and long-term stability remains limited and heterogeneous. Given the variability in study protocols, HA formulations, and follow-up durations, the available data should therefore be interpreted with caution.
